# Cross-β helical filaments of Tau and TMEM106B in gray and white matter of multiple system tauopathy with presenile dementia

**DOI:** 10.1007/s00401-023-02563-3

**Published:** 2023-03-23

**Authors:** Md. Rejaul Hoq, Sakshibeedu R. Bharath, Grace I. Hallinan, Anllely Fernandez, Frank S. Vago, Kadir A. Ozcan, Daoyi Li, Holly J. Garringer, Ruben Vidal, Bernardino Ghetti, Wen Jiang

**Affiliations:** 1 Department of Biological Sciences, Markey Center for Structural Biology, Purdue University, West Lafayette, IN 47906 USA; 2 Department of Pathology and Laboratory Medicine, Indiana University School of Medicine, 635 Barnhill Dr., MSB A136, Indianapolis, IN 46202 USA; 3 Stark Neurosciences Research Institute, Indiana University School of Medicine, Indianapolis, IN 46202 USA

In neurodegenerative diseases and aging, the microtubule-associated protein tau (MAPT) and the transmembrane lysosomal protein 106B (TMEM106B) become misfolded in different cell types and give rise to intracellular inclusions [3, 4]. The latter are made of amyloid filaments whose structures are being studied at the molecular level by cryogenic electron microscopy (cryo-EM). The nature of intracellular tau aggregates is determined by the participating tau isoforms and the structure of the amyloid filament(s) [1, 4]. TMEM106B aggregates, as discovered using cryo-EM, are composed of amyloid filaments that originate from the carboxy terminus of TMEM106B [3].

In the brain, the gray matter differs from the white matter for the cell types and the quantity of myelin. The gray and white matter both contain astrocytes, oligodendrocytes, and microglia. The gray matter contains nerve cell bodies, dendrites, axons, and synaptic terminals whereas the white matter contains axons as the only nerve cell component. The structure of tau and TMEM106B amyloid filaments present in the gray matter has been unveiled in several neurodegenerative diseases using cryo-EM; however, whether tau and TMEM106B filaments from the gray and white matter have the same fold is unknown.

Neuropathologic, biochemical, genetic, and cryo-EM methods were used to study the gray and white matter from the frontal lobes of two individuals affected by multiple system tauopathy with presenile dementia (MSTD) (Supplementary Figs. 1–6). MSTD is a neurodegenerative disease caused by the *MAPT* intron 10 mutation + 3, which disrupts a stem-loop structure in the mRNA and leads to the presence of mainly four repeat (4R) tau isoforms in neurons and glia [2, 5–7]. Tau inclusions labelled by antibodies to phosphorylated tau and to 4R have different shapes in neurons, astrocytes, and oligodendrocytes. In the gray matter, tau inclusions are present in neurons and glia including tufted astrocytes, astrocytic plaques, and oligodendroglia with coiled bodies. In the white matter, tau inclusions are seen in oligodendrocytes with numerous coiled bodies and astrocytes (Supplementary Fig. 1, 2). TMEM106B inclusions in the gray and white matter were labelled with anti-TMEM239 (residues 239–250). In the gray and white matter, numerous intracellular inclusions were present mostly in the cell bodies and processes of astrocytes (Supplementary Fig. 2).

Using a multidisciplinary approach, we characterized tau and TMEM106B amyloid filaments from the gray and the white matter. Relative to tau, we have determined that both areas contain filaments that have the AGD type 2 fold with a four-layered ordered structure accommodating amino acids 279–381 of tau, packing two protofilaments with C_2_ symmetry (Fig. 1). The AGD type 2 fold was previously shown for filaments obtained from the gray matter of these two cases [4]. Using Western blot analysis, tau from the gray and white matter of the two individuals had identical biochemical profiles, by immunogold labelled negative stain electron microscopy had indistinguishable filaments and had equal seeding of tau misfolding in a biosensor cell line for tau aggregation (Supplementary Fig. 3).

**Fig. 1:**
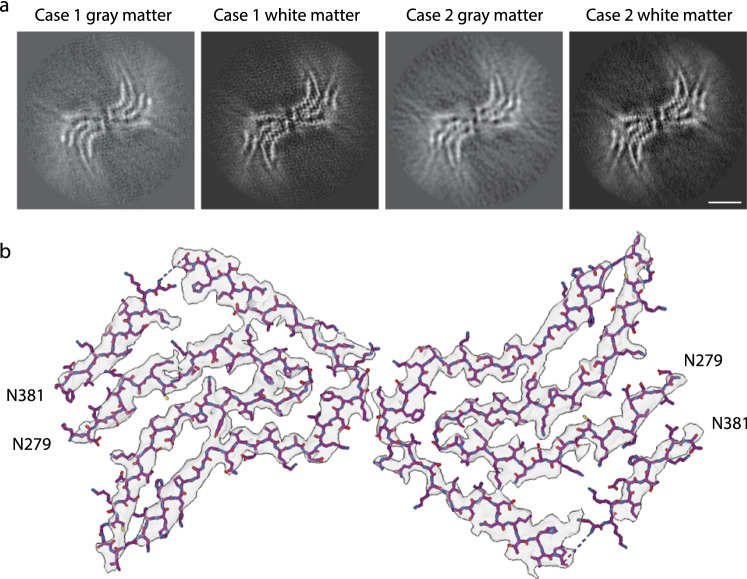
*Cryo-EM structure of tau filaments.* **a** Cryo-EM maps for tau filaments from gray and white matter from MSTD cases #1 and #2 showing the AGD type 2 filaments. **b** Cryo-EM density map and atomic model of AGD type 2 filaments. Scale bar 5 nm

We also determined that TMEM106B amyloid filaments were identical in the gray and white matter. The TMEM106B protofilament core spans residues 120–254 and consists of 17 β-strands. Filaments were made of one or two protofilaments (Fig. 2). When a filament was made by two protofilaments, these were identical. Densities corresponding to four asparagine (N) glycosylation sites (N145, N151, N164, and N183) were observed (Fig. 2).

**Fig. 2:**
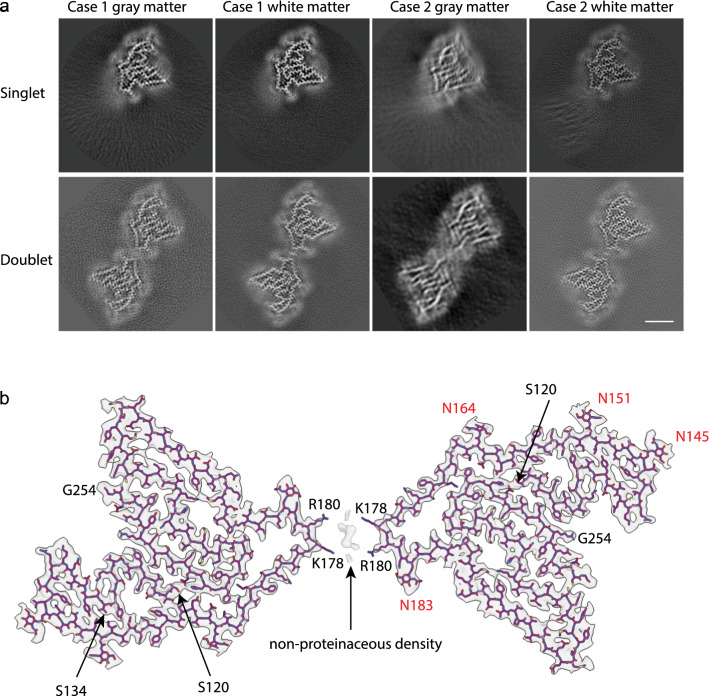
*Cryo-EM structure of TMEM106B filaments.* **a** Cryo-EM maps for TMEM106B filaments from gray and white matter from MSTD cases #1 and #2 showing filaments made of a single protofilament and filaments comprising two protofilaments. Scale bar: 5 nm. **b** Cryo-EM density map and atomic model of the doublet form of a TMEM106B filament. The N-terminus (S120) and C-terminus (G254) are indicated in black. Asn (N) glycosylated residues are indicated in red. K178 and R180 are involved in the binding of an unknown cofactor. Position of Ser134 (S134) that is replaced in one allele by Asn (S134N) in Case #1 is indicated

By whole exome sequencing, it was determined that case #1 was heterozygous for a polymorphism at codon 134 (S134N) of *TMEM106B*. The encoded residue is inside the protofilament core; however, by cryo-EM, it was not possible to establish whether filaments contained serine or asparagine at position 134 of the core, due to the similarities in the electrodensity of the two residues.

In MSTD, the protofilaments extracted from the gray matter were shown for the first time to have the same fold as those extracted from the white matter for both tau and TMEM106B, respectively. Whether in MSTD, there is a relationship between tau and TMEM106B filaments in the pathogenesis of this disorder remains to be determined. Furthermore, additional studies of other neurodegenerative diseases are needed to establish whether amyloid filaments deriving from other proteins extracted from the gray and white matter would have identical folds like in MSTD. If future studies show that in genetically determined neurodegenerative diseases other amyloid proteins have identical filament cores in the gray and white matter, it might suggest that a common mechanism of misfolding takes place regardless of cell composition in different anatomical areas.

## Supplementary Information

Below is the link to the electronic supplementary material.

Supplementary file1 (PDF 4222 KB)

## Data Availability

Cryo-EM maps have been deposited in the Electron Microscopy Data Bank (EMDB) under accession numbers EMD-25995 and EMD-28943. Refined atomic models have been deposited in the Protein Data Bank (PDB) under accession numbers 7TMC and 8F9K. Mass spectrometry raw data are available at MassIVE under accession number MSV000090845. Whole-exome sequencing data have been deposited in the National Institute on Aging Alzheimer’s Disease Data Storage Site (NIAGADS; https://www.Niagads.org), under accession number NG00107.
